# Supplementary material for: No more than three PlpE non-overlapping epitopes trigger significant antibody production in individuals vaccinated with the Pasteurella multocida epitope-chimeric proteins
Source: Microbiol Spectr. 2026 Feb 3;14(3):e02878-25. doi: 10.1128/spectrum.02878-25 (PMC12955485; doi:10.1128/spectrum.02878-25)
Supplement: Supplemental material — Tables S1 to S3; Fig. S1 and S2. [file spectrum.02878-25-s0001.docx]

Table S1 Primers used in the preparation of PlpE chimeric proteins with other proteins

| Primer name | Sequence (5' to 3') | Construct generated |
| --- | --- | --- |
| 28a-F | GATCCGGCTGCTAACAAAG | Linear plasmid |
| 28a-R | CATGGTATATCTCCTTCTTAAAGTT |  |
| PlpE-VP60P1 | taagaaggagatataccatgTGTAGCGGTGGTGGCGGT | PlpE fragment upstream |
| PlpE-VP60P2 | cctgccggactATAGTTTTCTTCTATTTTTTTATAATCTGATGA |  |
| PlpE-VP60P3 | gaaaactatAGTCCGGCAGGTCTGCTGA | VP60 P domain fragment |
| PlpE-VP60P4 | ccaccgctacaAACATAGCTGAAACCATTGGTGG |  |
| PlpE-VP60P5 | agctatgttTGTAGCGGTGGTGGCGGT | PlpE fragment downstream |
| PlpE-VP60P6 | gctttgttagcagccggatcTTAATAGTTTTCTTCTATTTTTTTATAATCTGATGA |  |
| PlpE-BcfA-1 | taagaaggagatataccatgTGTAGCGGTGGTGGCGGT | PlpE fragment |
| PlpE-BcfA-2 | gcgcgatcgtATCTTCTTGATGGTAAGTTGCAGTTAG |  |
| PlpE-BcfA-3 | tcaagaagatACGATCGCGCGCGTCGAT | BcfA fragment |
| PlpE-BcfA-4 | gctttgttagcagccggatcTTAGCTTCCCAGCAGGCCGCC |  |
| PlpE-PtfA-1 | tgcatcatcatcatcatcatTGTAGCGGTGGTGGCGGT | PlpE fragment |
| PlpE-PtfA-2 | gcgtaaaATCTTCTTGATGGTAAGTTGCAGTTAG |  |
| PlpE-PtfA-3 | ccatcaagaagatTTTACGCTAATTGAATTAATGATTGTCA | PtfA fragment |
| PlpE-PtfA-4 | cagtggtggtggtggtggtgTGCACAAAATCCTGCTGGG |  |

Note: the sequences in the primers that are homologous to the vector are denoted in lowercase letters.

Table S2 Primers used in the preparation of recombinant proteins chimeric with 4 PlpE non-overlapping epitopes

| Primer name | Sequence (5' to 3') | Construct generated |
| --- | --- | --- |
| 28a-F | GATCCGGCTGCTAACAAAG | linear plasmid |
| 28a-R | CATGGTATATCTCCTTCTTAAAGTT |  |
| ABCD-1 | taagaaggagatataccatgTGTAGCGGTGGTGGCGGT | upstream PlpE fragment containing epitope A, B, C, D |
| ABCD-2 | gacctgccggactTACATTATTACTCGCATTTCCCCA |  |
| ABCD-3 | taatgtaAGTCCGGCAGGTCTGCTGA | VP60 P domain fragment |
| ABCD-4 | ccaccgctacaAACATAGCTGAAACCATTGGTGG |  |
| ABCD-5 | agctatgttTGTAGCGGTGGTGGCGGT | downstream PlpE fragment containing epitope A, B, C, D |
| ABCD-6 | gctttgttagcagccggatcTTATACATTATTACTCGCATTTCCCCA |  |
| ABEF-1 | taagaaggagatataccatgTGTAGCGGTGGTGGCGGT | upstream PlpE fragment containing epitope A, B, |
| ABEF-2 | ctcgcTTCTTTGGATGTGTCATGAGAAGAC |  |
| ABEF-3 | gacacatccaaagaaGCGAGTAATAATGTAGATAAAGATAATGTTACG | upstream PlpE fragment containing epitope E, F, |
| ABEF-4 | cctgccggactATAGTTTTCTTCTATTTTTTTATAATCTGATGA |  |
| ABEF-5 | gaaaactatAGTCCGGCAGGTCTGCTGA | VP60 P domain fragment |
| ABEF-6 | ccaccgctacaAACATAGCTGAAACCATTGGTGG |  |
| ABEF-7 | agctatgttTGTAGCGGTGGTGGCGGT | downstream PlpE fragment containing epitope A, B |
| ABEF-8 | ctcgcTTCTTTGGATGTGTCATGAGAAGAC |  |
| ABEF-9 | gacacatccaaagaaGCGAGTAATAATGTAGATAAAGATAATGTTACG | downstream PlpE fragment containing epitope E, F |
| ABEF-10 | gctttgttagcagccggatcTTAATAGTTTTCTTCTATTTTTTTATAATCTGATGA |  |
| CDEF-1 | taagaaggagatataccatgTCAATGTCTTCTCATGACACATCCA | upstream PlpE fragment containing epitope C, D, E, F |
| CDEF-2 | cctgccggactATAGTTTTCTTCTATTTTTTTATAATCTGATGA |  |
| CDEF-3 | gaaaactatAGTCCGGCAGGTCTGCTGA | VP60 P domain fragment |
| CDEF-4 | gtgtcatgagaagacattgaAACATAGCTGAAACCATTGGTGG |  |
| CDEF-5 | TCAATGTCTTCTCATGACACATCCA | downstream PlpE fragment containing epitope C, D, E, F |
| CDEF-6 | gctttgttagcagccggatcTTAATAGTTTTCTTCTATTTTTTTATAATCTGATGA |  |

Note: the sequences in the primers that are homologous to the vector are denoted in lowercase letters.

Table S3 Effective epitope ratio for each PlpE non-overlapping epitope and total number of PlpE effective epitopes in different chimeric proteins

| Effective epitope | Chimeric protein with Single epitope | Chimeric protein with 2 epitopes | | | | | | Chimeric protein with 3 epitopes | |
| --- | --- | --- | --- | --- | --- | --- | --- | --- | --- |
|  |  | AC | AE | CE | BD | BF | DF | BDF | ACE |
| A | 0.4 | 0.5 | 0.7 |  |  |  |  |  | 0.7 |
| B | 0.4 |  |  |  | 0 | 0 |  | 0.5 |  |
| C | 0.2 | 0.1 |  | 0.5 |  |  |  |  | 0.4 |
| D | 0.4 |  |  |  | 0 |  | 0 | 0.2 |  |
| E | 0.6 |  | 0.9 | 0.9 |  |  |  |  | 1 |
| F | 0.1 |  |  |  |  | 0 | 0.2 | 0.2 |  |
| Total | - | 0.6 | 1.6 | 1.4 | 0 | 0 | 0.2 | 0.9 | 2.1 |

Note： The effective epitope ratio = The number of vaccinated individuals in whom the epitope triggers a significant antibody response/the total number of vaccinated individuals. The total number of PlpE effective epitopes = Sum of the effective epitope ratios of the PlpE epitopes contained in the chimeric protein.

Table S3 (continued) Effective epitope ratio for each PlpE non-overlapping epitope and total number of PlpE effective epitopes in different chimeric proteins

| Effective epitope | Chimeric protein with 4 epitopes | | | Chimeric protein with 6 epitopes | | | |
| --- | --- | --- | --- | --- | --- | --- | --- |
|  | ABCD | ABEF | CDEF | PlpE-VP60P | PlpE-BcfA | PlpE-PtfA | PlpE |
| A | 0.3 | 0.4 |  | 0.1 | 0.3 | 0.1 | 0.8 |
| B | 0.2 | 0.9 |  | 0.1 | 0 | 0 | 0.7 |
| C | 0.3 |  | 0 | 0 | 0.1 | 0 | 0.1 |
| D | 0.5 |  | 0.1 | 0.1 | 0 | 0 | 0.2 |
| E |  | 0.3 | 0.6 | 0.5 | 0.2 | 0.1 | 0.8 |
| F |  | 0 | 0.1 | 0.4 | 0.3 | 0 | 0 |
| Total | 1.3 | 1.6 | 0.8 | 1.2 | 0.9 | 0.2 | 2.6 |

Note： The effective epitope ratio = The number of vaccinated individuals in whom the epitope triggers a significant antibody response/the total number of vaccinated individuals. The total number of PlpE effective epitopes = Sum of the effective epitope ratios of the PlpE epitopes contained in the chimeric protein.


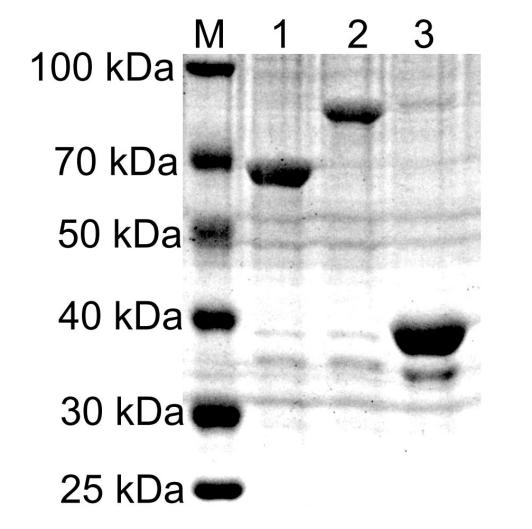
Fig. S1 Recombinant proteins chimerized with PlpE dominante region and other proteins. Lanes M and 1 to 3 show the marker, PlpE-VP60P, PlpE-BcfA, PlpE-PtfA.


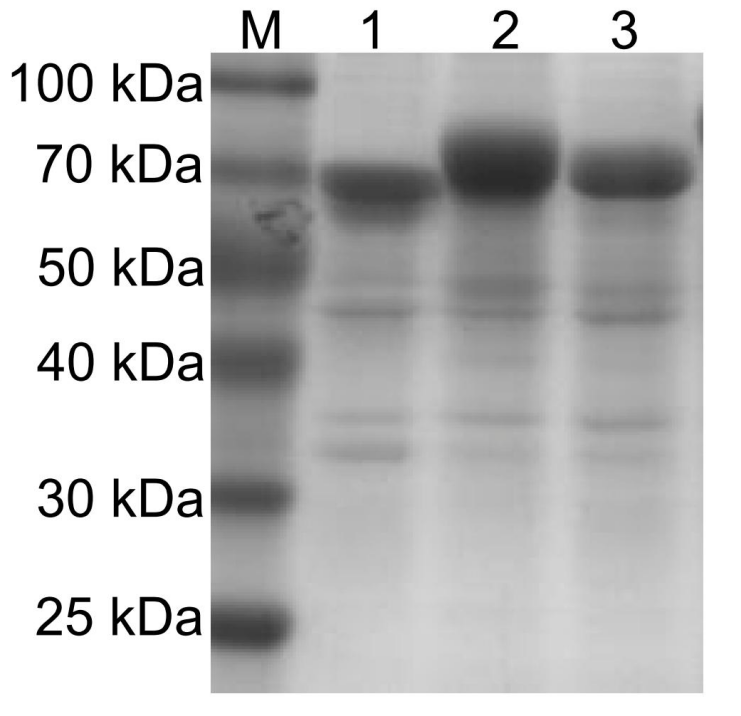


Fig. S2 Recombinant chimeric proteins with 4 PlpE non-overlapping epitopes. Lanes M and 1 to 3 show the marker, recombinant protein chimeric with PlpE epitope A, B, C, and D (named ABCD), with PlpE epitope A, B, E, and F (named ABEF), with PlpE epitope C, D, E, F (named CDEF).
